# Optimizing PCR Detection of West Nile Virus from Body Fluid Specimens to Delineate Natural History in an Infected Human Cohort

**DOI:** 10.3390/ijms20081934

**Published:** 2019-04-19

**Authors:** Rodion Gorchakov, Bonnie E. Gulas-Wroblewski, Shannon E. Ronca, Jeanne C. Ruff, Melissa S. Nolan, Rebecca Berry, R. Elias Alvarado, Sarah M. Gunter, Kristy O. Murray

**Affiliations:** 1 Department of Pediatrics, National School of Tropical Medicine, Baylor College of Medicine and Texas Children’s Hospital, Houston, TX 77030, USA; rodion.gorchakov@gmail.com (R.G.); Bonnie.Gulas-Wroblewski@bcm.edu (B.E.G.-W.); ronca@bcm.edu (S.E.R.); jruff@bcm.edu (J.C.R.); rberry@bcm.edu (R.B.); Rojelio.Alvarado@bcm.edu (R.E.A.); sm22@bcm.edu (S.M.G.); 2 Department of Wildlife and Fisheries Sciences, Texas A&M University, College Station, TX 77843, USA; 3 University of South Carolina, Arnold School of Public Health, Columbia, SC 29208, USA; MSNOLAN@mailbox.sc.edu

**Keywords:** West Nile virus, virus shedding, PCR, prolonged detection, saliva, urine, whole blood, semen, Houston West Nile cohort

## Abstract

West Nile virus (WNV), a mosquito-borne arbovirus, remains a major global health concern. In this study, we optimized PCR methods then assessed serially-collected whole blood (WB), urine (UR), saliva, and semen specimens from a large cohort of WNV-positive participants to evaluate the natural history of infection and persistent shedding of WNV RNA. Viral RNA extraction protocols for frozen WB and UR specimens were optimized and validated through spiking experiments to maximize recovery of viral RNA from archived specimens and to assess the degradation of WNV RNA in stored UR specimens. The resultant procedures were used in conjunction with PCR detection to identify WNV-positive specimens and to quantify their viral loads. A total of 59 of 352 WB, 10 of 38 UR, and 2 of 34 saliva specimens tested positive for WNV RNA. Although a single semen specimen was positive 22 days post onset, we could not definitively confirm the presence of WNV RNA in the remaining specimens. WNV RNA-positive UR specimens exhibited profound loss of viral RNA during storage, highlighting the need for optimal preservation pre-storage. This study provides optimized methods for WNV RNA detection among different fluid types and offers alternative options for diagnostic testing during the acute stages of WNV.

## 1. Introduction

West Nile virus (WNV), a positive-stranded RNA virus in the Japanese encephalitis serocomplex, is a mosquito-borne flavivirus of significant world-wide public health concern. Isolated in Uganda in 1937, the virus is endemic in Africa, parts of Eurasia, the Americas, and Australia [1,2,3]. First detected in the United States in New York in 1999, WNV rapidly spread throughout the continental states by 2002 and to other regions of the Americas in subsequent years. Since its initial discovery in New York state, WNV has been responsible for thousands of clinical cases in the United States, with a 3%–7% case fatality rate [4]. The costs associated with acute and long-term medical care for hospitalized WNV cases have been estimated at $56 million per year [5].

Infections are typically asymptomatic or subclinical, although about 20% of WNV cases develop mild, flu-like symptoms that last between two and seven days. In less than 1% of cases, WNV infection progresses to neuroinvasive disease, characterized by meningitis, encephalitis, and/or acute flaccid paralysis [1,2,3]. In a subset of patients, neurologic complications persist years after acute illness, especially for those who initially experienced West Nile encephalitis [6]. Chronic neurologic symptoms include abnormal movement conditions, neurocognitive disorders, behavioral disorders, functional impairment, and retinopathy [6,7,8,9,10,11,12,13,14]. Similarly, there is mounting evidence of viral persistence in body fluids long after the acute phase of WNV disease [15,16,17,18,19,20,21,22]. Previous investigations of chronic neurologic conditions attributed to WNV infections have generally neglected to analyze long-term persistence of WNV RNA in body fluids and tissues [6,7,8,9,10,11,12,13,14]. Studies that use archived specimens to assess chronic shedding of WNV RNA may be hindered by differential decomposition of viral material in various body fluids during storage. Identification of prolonged persistence of WNV RNA in various body fluids is crucial to understanding the natural progression of the disease, identifying comprehensive diagnostic sampling protocols, and formulating long-term medical support plans for patients infected with WNV.

To address this need, we optimized extraction protocols for WNV RNA from whole blood (WB) and urine (UR) for PCR detection in order to evaluate the persistence of WNV RNA in a variety of serially-collected body fluid specimens from a large cohort (n = 184) of WNV-infected study participants in Southeast Texas.

## 2. Results

### 2.1. Optimization of WNV RNA Recovery from Archived WB and UR Specimens

**Figure 1:**
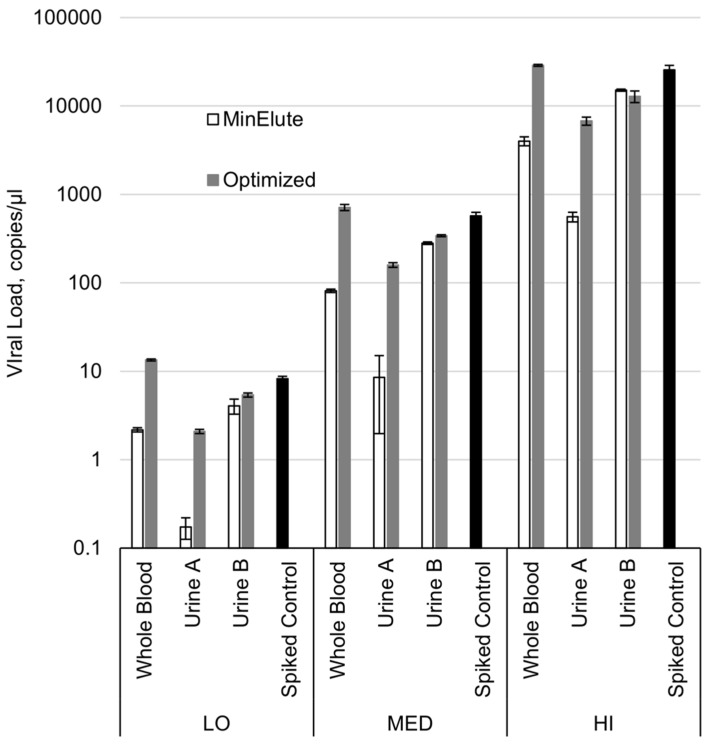
West Nile virus (WNV) RNA recovery from whole blood and urine specimens with the original protocol and after optimization. LO, low spiking load; MED, medium spiking load; HI, high spiking load. Bars indicate one standard deviation.

Prior to testing of the archived body fluid specimens from the Houston West Nile Cohort (HWNC), our methods for RNA extraction from frozen WB and UR specimens were evaluated for efficacy in the recovery of WNV RNA. Controlled spiking of specimens from WNV-negative subjects validated that the original extraction protocol conferred a limited recovery of WNV RNA from WB specimens (19% average across the three spiking loads), while a complete restoration of WNV RNA recovery from WB was attained with a slightly adjusted procedure (Figure 1, Tables S1 and S2). As similarly described with Zika virus (ZIKV) in Gorchakov et al., there was variation in WNV RNA recovery from UR specimens of two different negative donors [23] (Figure 1, Table S2). UR from donor A significantly hindered RNA extraction via the original method, with only a 2% average recovery across the three spiking loads. Incorporating the Urine Conditioning Buffer (UCB) treatment into the protocol restored the WNV RNA detection from donor A’s UR to within two Ct (cycle threshold) from the expected value. In the case of UR from donor B, both extraction methods exhibited an acceptable recovery of WNV RNA, with all replicates within one Ct from the expected value and the average RNA recovery over 50%. Moreover, utilization of UCB increased the qualitative sensitivity of the method due to the higher volume (in our procedure, five-fold: 1 mL vs. 200 µL) of the UR specimen used for extraction. For example, while the average recovery in terms of estimated cp/µl concentration in the UR B specimens extracted with the original and the optimized procedures were 52% and 58%, respectively, the actual Ct values recovered by the optimized method were lower than those produced by the original method by an average of 2.4 units (Table S2).

### 2.2. WNV RNA Recovery from WB from HWNC

A total of 352 WB specimens from 184 HWNC participants with available WB samples were tested for the presence of WNV RNA, with a mean time of collection of 1612 days post onset (DPO) (range, 4–3228 DPO). A total of 59 of the 352 WB specimens (16.8%) tested positive for WNV RNA, collected from 19 different subjects within 126 DPO. Five subjects with only negative specimens had the first available specimen collected within 150 DPO (range 15–705 DPO).

The viral load in WNV RNA-positive WB specimens was relatively low for all specimens: below the lower limit of quantification for six of the specimens and ranging between 1.0 and 21.2 cp/μl for viral loads that could be quantified. A trend of decreasing WNV RNA concentration was demonstrated in specimens consecutively-collected from the same patient over time. 

**Figure 2:**
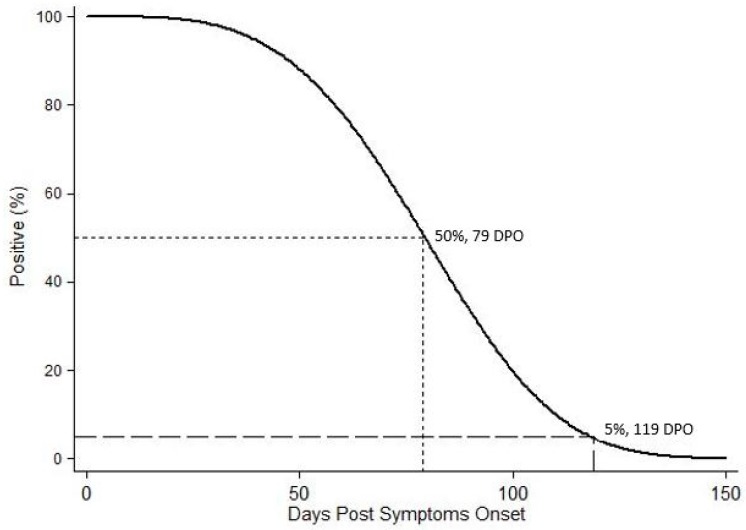
Weibull distribution model of the time to loss of WNV RNA detection in whole blood specimens.

When estimating the time to loss of WNV RNA detection in WB, temporal data for the thirteen subjects with multiple WB specimens positive for WNV RNA were interval-censored, while those for the remaining six subjects with single positive specimens were right censored. The time data for the 165 subjects with only negative WB specimens was left-censored for this analysis. Weibull distribution modeling of the time to loss of WNV RNA detection in WB was calculated. The median time for loss was at 79 DPO with the 95th percentile placed at 119 DPO (Figure 2).

### 2.3. WNV RNA Recovery from UR, Saliva, and Semen from 2018 WNV Cohort Participants

**Figure 3:**
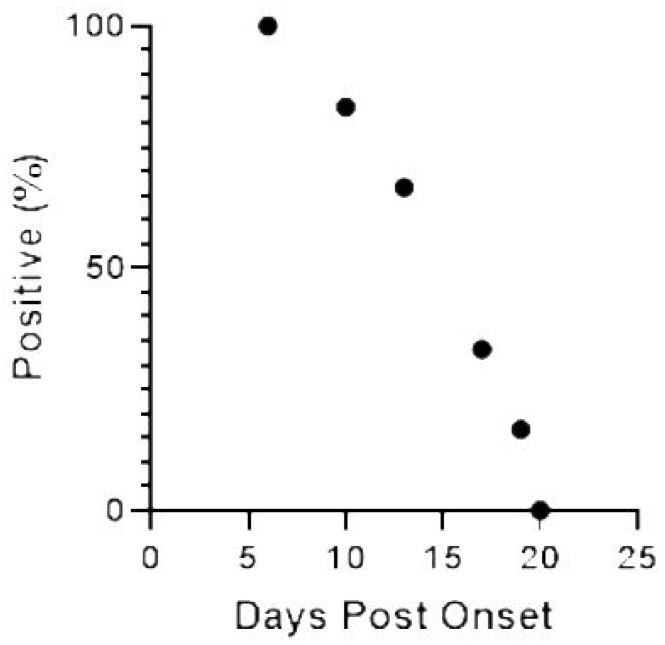
Viruria detected over time for urine collected from 2018 cohort participants with confirmed WNV infection.

Nine acutely-infected participants were prospectively enrolled in the cohort during the 2018 West Nile season, from which a total of 38 UR specimens were collected. The median time of UR specimen collection was 24 DPO (range, 4–144 DPO), and the first collection available for each subject ranged from 4–21 DPO. A total of 10 UR specimens tested positive for WNV RNA, all collected within the first 18 DPO and representing samples collected from 7 participants. The two remaining participants did not have detectable virus in their urine at enrollment (18 and 21 DPO) (Figure 3). The viral load in WNV RNA-positive UR specimens analyzed before freezing ranged between 0.27 and 1.03 cp/µl. In four cases, when UR and WB were collected on the same dates, the viral load in WNV RNA-positive UR specimens was higher than the viral load in WB specimens, with a mean difference of 0.42 cp/μl (range, 0.19–0.84 cp/μl).

We evaluated the effects of freezing on RNA detection for four of the aforementioned WNV RNA-positive UR specimens. Initial viral loads for these UR samples pre-freezing ranged from below the level of quantification (0.2 cp/μl) to 1.03 cp/μl. Aliquots of the fresh UR specimens were frozen at −80 °C for at least 130 days, then thawed, extracted, and run by PCR to assess reproducibility of viral RNA detection post-freezing. Following 161, 177, and 198 days of storage at −80 °C, three of the previously-positive UR specimens from two different participants tested negative by PCR using our optimized method of extraction. In two cases, these values were 0.6 and 0.19 cp/μl less than the viral loads calculated for the same UR specimen prior to freezing. One of the four UR specimens exhibited a higher viral load after 199 days of freezing (0.39 cp/μl), when compared to the viral load from the original collection date (BLQ). 

Thirty-four saliva specimens were collected from the 2018 cohort participants between 4 and 108 DPO and tested for WNV RNA. Of these specimens, two tested positive from a single participant, collected 4 DPO and 9 DPO. Viral loads for both WNV RNA-positive saliva specimens were too low to determine.

Four semen specimens from three participants were screened for WNV RNA. Specimens were collected 9–34 DPO (median, 20.5 DPO), and three were negative in accordance with the Ct unit cutoff value defined in our protocols. However, a single semen specimen collected on 22 DPO met the criteria for a positive sample, although it was very close to the defined cutoff (39.7 vs. a cutoff of 40.0).

## 3. Discussion

Our comparison of extraction methodologies using spiked specimens demonstrates that small adjustments to QIAamp MinElute Virus Spin Kit protocol facilitate the complete recovery of WNV RNA for PCR detection from “as is” WB specimens, despite undergoing a freeze-thaw cycle, similar to what is already published for ZIKV [23]. Although ZIKV and WNV are closely related, it is always prudent to confirm methodologies with the specific viral species of interest. Maximum recovery of WNV RNA from UR specimens also benefited from alterations to standard extraction protocols: The addition of UCB from Zymo Research for defrosting UR specimens was crucial for optimum viral RNA extraction from frozen UR, as well as for increasing the sensitivity of the method for use with higher UR volumes (Figure 1, Table S2). Optimizing extraction protocols for WNV RNA from WB and UR for PCR detection was essential before advancing to analysis of the HWNC specimens and assessing the efficacy of WNV RNA recovery from frozen UR specimens. Complex specimen matrices, particularly of UR, present a multitude of challenges for the extraction of complete RNA, free from any impurities that might affect downstream reactions [24,25,26,27,28]. We were only able to proceed with testing the bank of archived specimens collected longitudinally from HWNC participants after validating the efficacy of our methods for the full recovery of contamination-free WNV RNA from frozen WB specimens.

Included in our analysis were 352 WB specimens collected in the range from disease onset to 8.8 years post WNV infection, of which 59 were positive for WNV RNA with a low level of target RNA quantity. Consistent with previous studies for WNV and other flaviviruses, such as dengue virus and ZIKV, all WNV-positive specimens detected were collected within 126 DPO [17,18,22,23,29,30,31,32]. Extended duration of WNV RNA shedding was substantiated by the Weibull distribution modeling of the time to loss of WNV RNA detection in WB, which calculated the median time to loss of viral RNA detection at 79 DPO and the time when 95% of participants tested would be WNV-negative at 119 DPO (Figure 2). These values accurately fit the actual data from our study as well as previously published results. However, this analysis was limited by the paucity of post onset WNV-positive archived specimens and the constraint of having to right-, left-, or interval-censor all of the observations. Nevertheless, an encouraging outcome of this investigation is that none of the WB specimens collected after 126 DPO up to 8.8 years post onset were positive for WNV RNA, even in subjects with West Nile encephalitis at acute onset and/or those suffering from long-term effects of the disease [6,7,10,11,12,33]. This finding suggests that despite the possibility of persistent WNV infection in some of our subjects [15,16,19,20,22,34,35,36,37,38,39], the virus is unlikely to circulate throughout the body during this time or remains below the level of detection in blood. 

A possible, although unlikely, restriction of our study was the potential for WNV RNA degradation in WB specimens during storage. We anticipate this effect to be minimal because, unlike the harsh chemical composition of UR, the serum fraction of WB is a perfectly formulated buffer and widely used for the cryopreservation of viral specimens. In fact, eight of the WB specimens that we identified as WNV-positive were in frozen storage for almost six years and one had been archived for nearly 11 years.

We recovered WNV RNA from 10 of the 38 UR specimens, from 7 participants up to 18 DPO, adding further to the burgeoning evidence for detection of WNV RNA in UR in the acute stage of the disease [18,21,22,40] and after the date of onset [15,19,20,22]. In four of these WNV RNA-positive UR specimens, the viral load recovered was higher than that evidenced in concurrently-collected WB specimens from the same participants. Consequently, diagnostic tests for WNV participants should incorporate UR sampling into their standard protocols in order to improve the probability of WNV detection in the early stages of infection. 

It was recently demonstrated that low levels of ZIKV in spiked urine specimens are susceptible to diminished detection by PCR after storage for 10 and 30 days at −80 °C, which was reversed with the addition of preservatives before storage [27]. Alternately, incorporation of the UCB method into UR extraction protocols provided excellent detection of low levels of ZIKV RNA from UR specimens archived for 9–10 months [23,32]. Since the extreme duration of UR specimen storage may prove a major limiting factor in the detection of WNV RNA in longitudinally-collected UR specimens, we examined the possible effects of freezing on the recovery of viral RNA in WNV-positive UR specimens. Following only 161, 177, and 198 days of storage at −80 °C, three of the previously-positive UR specimens from two different participants tested negative by PCR using our optimized method of extraction. Viral loads were calculated for the remaining WNV RNA-positive UR specimens re-extracted post-freezing, and all but one exhibited viral loads lower than those recovered from the same UR specimen analyzed pre-freezing. Future work should focus on methodologies for minimizing the degradation of WNV RNA in UR during storage, and investigations of chronic WNV RNA-shedding in UR should acknowledge the limitation of RNA recovery from specimens stored for prolonged periods. 

Two of the 34 saliva specimens tested were positive for WNV RNA. No previous reports exist for the presence of WNV RNA in human saliva, though viral RNA has been recovered from the saliva of participants infected with ZIKV [41,42] and dengue virus [43,44]. The efficacy of using saliva as alternative or complimentary specimens for molecular diagnosis of WNV in its acute stages should be further explored, especially as the collection of such specimens is easier and less invasive than those of other body fluids, and more readily facilitates the testing of children and of participants providing specimens via self-collection. Future evaluation of the potential, however unlikely, for WNV RNA shed in saliva to reach infectious levels would be important to understand the risk of transmission.

The presence of viral RNA in semen and its potential to contribute to the sexual transmission of arboviruses has been well-established in the case of ZIKV [45] and described for Japanese encephalitis virus in experimentally-infected boars [46]. Despite our inability to recover sufficient WNV RNA to confirm the presence of viral RNA in three semen specimens, the positive (albeit close to the cut-off Ct value) fourth specimen collected from a single participant in the 2018 WNV cohort is notable. The potential for WNV RNA to be shed in semen should not be discounted, and WNV surveillance efforts should include semen in their sampling protocols whenever possible. 

Optimizing detection of WNV in body fluids is crucial for research and diagnostics. Although the use of archived UR specimens in the investigation of chronic viral shedding may be limited by storage time, WB specimens prove to be valuable specimens for the prolonged detection of WNV RNA. The persistence of WNV in WB of affected individuals was confirmed by this study, which also facilitated calculation of the best estimate to date for the long-term presence of WNV in systemic circulation within infected participants. In future studies, it will be important to determine how WNV RNA persistence correlates to immunity and complications of disease. 

## 4. Materials and Methods 

### 4.1. Study Population, Ethics Statement, and Specimen Collection

The HWNC was initiated in Texas in 2002. WNV-infected participants were identified through surveillance by the city of Houston and Harris County local health departments. WNV-positive cases were also located through blood donation screenings performed by the Gulf Coast Regional Blood Center. All detected WNV cases were invited to participate in the WNV longitudinal cohort study. No exclusion criteria were defined for age, gender, race, or ethnicity. Several control subjects, who did not have clinical or diagnostic evidence of WNV infection, were also enrolled in the study. Consents were obtained from cases and controls willing to participate via approved IRB protocols. All participants provided written consent to participate. Over 300 participants were enrolled in the HWNC between 2002 and 2018. The study protocol and consent form were approved by the University of Texas Health Science Center at Houston Committee for the Protection of Human Subjects (HSC-SPH-03-039 and HSC-SPH-18-0333, approved 14 May 2018) and Baylor College of Medicine Institutional Review Board (H-30533, approved 11 July 2018).

Upon enrollment into the HWNC cohort, an extensive interview was conducted to collect baseline characteristics ranging from demographic and health history data to physical functioning index and mental state score. An abbreviated questionnaire was used during follow-up interviews at six months intervals to keep track of relevant clinical symptoms. 

During initial and follow-up visits with the HWNC participants, serum, plasma, WB, and UR specimens were collected, aliquoted by 1 mL, and stored at −80 °C. For participants enrolled in 2018, saliva and semen were also collected and stored. All specimens collected in 2018 were tested for WNV RNA within 7 days of collection or as otherwise indicated. 

### 4.2. Optimization of WNV RNA Extraction from WB and UR Specimens

Fresh WB and UR specimens from WNV-negative donors were spiked with WNV virus, frozen at −80 °C for 24 h, and then processed for RNA extraction. The interval of freezing served to mimic the effect of storage temperature and freeze/thaw cycle of the archived specimens. The viral specimen used for spiking was a cell culture supernatant of Vero cells infected with WNV strain 2004Hou3, isolated from a squirrel in Houston, TX in 2004 [20]. Ten aliquots of 200 µL were prepared for each body fluid specimen: one unspiked control and triplicates for each of the three viral spiking loads, low (LO), medium (MED), and high (HI), spiked with 10 µL of the respective dilution of virus. Ten aliquots of 200 µL of AVE buffer (QIAGEN, Valencia, CA, USA), the elution buffer from the QIAGEN RNA extraction kit, was spiked accordingly as the reference buffer control, which was performed independently three times. For UR specimens, an additional set of 1 mL aliquots was prepared and spiked with 50 µL of diluted virus to achieve the same WNV concentration as above. 

Frozen spiked specimens were thawed, and RNA was extracted with a QIAamp MinElute Virus Spin kit (QIAGEN) directly by the manufacturer’s protocol with elution in 30 µL of AVE buffer. Optimized RNA extraction procedures for WB (packed cells fraction) and UR specimens were previously determined for this extraction kit [23]. Accordingly, the following adjustments were made to optimize RNA extraction from the WNV-spiked specimens. For WB specimens, incubation with Protease was extended to 30 min, initial specimen load on the column performed at 3000× *g*, and elution incubation done for 5 min at 56 °C. In the current study, 200 µL aliquots of WB samples were processed as is, without obtaining packed cells fraction and without increasing lysis volume, as in Gorchakov et al. For UR specimens, 70 µL of UCB (Zymo Research, Irvine, CA, USA) were added to the still-frozen 1 mL aliquots and then thawed with constant mixing. UR specimens were centrifuged at 3,000 × g for 15 min, pellets were dissolved in the QIAGEN kit lysis mix (25 µL Protease, 200 µL AVE, 200 µL AL, 5.6 µL carrier RNA), and processed by the manufacturer’s protocol starting with the Protease incubation step. RNA extraction from the HWNC WB and UR specimens was performed according to the optimal procedures described above.

### 4.3. Extraction of WNV RNA in Saliva and Semen Specimens

To screen the saliva and semen specimens of participants, RNA was extracted with a QIAamp MinElute Virus Spin kit (QIAGEN) directly by the optimized manufacturer’s protocol with elution in 30 µL of AVE buffer. This protocol was previously determined to be sufficient for extraction with other flaviviruses [23].

### 4.4. PCR Detection of WNV RNA in Specimens

In order to screen the extracted specimens for WNV RNA presence, 5 µL of extracted RNA from each specimen were used in a single 20 µL rRT–PCR reaction with TaqMan Fast Virus 1-Step Master Mix (Thermo Fisher Scientific, Waltham, MA, USA) on a ViiA 7 Real Time PCR System (Thermo Fisher Scientific) with a TaqMan WNV NS1 assay developed in our laboratory. The following controls were included in each rRT–PCR run: positive WNV RNA control, extraction negative control, and no template control. In addition, each extracted RNA specimen was tested in a separate rRT–PCR reaction with human RNase P TaqMan assay [47] as the internal positive control for nucleic acid extraction and absence of PCR inhibition. WNV-positive specimens and specimens from spiking experiments were run in duplicate alongside with the WNV RNA standard curve (transcribed RNA oligo) to estimate viral load in these specimens. Repeated runs of the standard curve specimens determined the lower limit of quantification to be equivalent to 1 copy of WNV RNA per microliter (cp/µl) in the original body fluid specimen for WB, saliva, and semen, but 0.2 cp/µl for UR due to the higher volume of the extracted specimen.

### 4.5. Statistical Methods

WNV RNA recovery from spiked negative specimens was considered acceptable if the specimen Ct values was within one Ct unit of the expected Ct value (from the spiked buffer control, Table S1) for over 80% of the replicates of the given specimen matrix. If the one Ct unit cutoff was not met, two Ct units difference from the expected Ct value for over 80% of the replicates was considered. The percent recovery for each level of viral load was calculated as follows:

(Average concentration in spiked matrix/average concentration in spiked buffer control) × 100%

The time of body fluid specimen collection was measured in DPO and corresponded to the difference in days between the date of specimen collection and either the date of West Nile disease symptoms onset or, for asymptomatic cases, the date of positive index blood donation. Subjects with at least one set of specimens collected in the same year as their WNV diagnosis, but without indication of acute infection at the time of collection (e.g., no WNV RNA- or WNV IgM-positive test), were excluded from these analyses. In the case of subjects with an unknown date of infection, the onset date was imputed as 15 September of the year of WNV diagnosis (no such imputation had to be done for the specimens with DPO <150). The time until loss of WNV RNA detection in a given body fluid was defined in DPO of the specimen with the negative WNV RNA test after the last positive WNV RNA test, so as to not exclude cases with possible intermittent shedding. All WNV cases were assumed to have positive tests for WNV RNA in both WB and UR at the time of disease onset. Data were right-censored for subjects with last available specimens that were still positive. Data were left-censored for subjects with only negative available specimens. Data were interval-censored if the time between the last positive and the first negative specimens was over one week for a given subject. STATA 12.1 software (StataCorp, College Station, TX, USA) was used to generate a Weibull distribution model in order to estimate the survival function for the time until loss of WNV RNA detection in WB as well as the median time and 95th percentile time for the outcome.

## Supplementary Materials

Supplementary materials can be found at https://www.mdpi.com/1422-0067/20/8/1934/s1.

## Abbreviations

BLQ	Below level of quantification
DPO	Days post onset
HWNC	Houston West Nile cohort
UCB	Urine Conditioning Buffer
UR	Urine
WB	Whole blood
WNV	West Nile virus
ZIKV	Zika virus
