# Supplementary material for: Optimizing PCR Detection of West Nile Virus from Body Fluid Specimens to Delineate Natural History in an Infected Human Cohort
Source: Int J Mol Sci. 2019 Apr 19;20(8):1934. doi: 10.3390/ijms20081934 (PMC6514913; doi:10.3390/ijms20081934)
Supplement: Supplementary file 1 [file ijms-20-01934-s001.pdf]

Supplementary Materials

Optimizing PCR Detection of West Nile Virus from Body Fluid Specimens to Delineate Natural History in an Infected Human Cohort

Table S1. Individual and average (expected) Ct values and respective concentrations of WNV RNA generated by repeated testing of the spiked reference control replicates.

	Set 1		Set 2		Set 3		Average		SD	
Replicates	Ct	cp/µl	Ct	cp/µl	Ct	cp/µl	Ct	cp/µl	Ct	cp/µl
LO 1	32.2	9.1	32.4	8.1	32.4	8.0	32.4	8.2	0.1	0.5
LO 2	32.3	8.6	32.4	7.6	32.3	8.8				
LO 3	32.4	8.2	32.4	8.0	32.5	7.6				
MED 1	26.2	646.0	26.5	497.3	26.4	538.3	26.4	571.6	0.1	56.0
MED 2	26.1	659.1	26.3	578.5	26.4	563.0				
MED 3	26.3	604.9	26.4	541.6	26.5	515.9				
HI 1	20.8	29,560.3	21.1	24,108.8	21.1	22,248.6	21.0	25,622.1	0.2	3181.2
HI 2	20.7	30,088.5	21.0	25,329.0	21.1	22,251.0				
HI 3	20.8	28,062.9	20.9	26,903.1	21.2	22,046.9				

SD, standard deviation; cp/µl, copies per microliter; LO, low spiking load; MED, medium spiking load; HI, high spiking load.

Table S2. Individual Ct values and percent recovery of WNV RNA in whole blood and urine specimens using original and optimized extraction protocols.

	**Rep 1 Ct**	**Rep 2 Ct**	**Rep 3 Ct**	**Expected Ct**	**#Rep within 1 Ct**	**% recovery**	**Average % recovery**
WB LO	34.2	34.4	34.2	32.4	0/3	26	19
WB MED	29.1	29.2	29.0	26.4	0/3	14	
WB HI	23.4	23.8	23.6	21.0	0/3	16	
WB optimized LO	31.6	31.6	31.7	32.4	3/3	163	133
WB optimized MED	26.2	26.0	26.0	26.4	3/3	125	
WB optimized HI	20.8	20.8	20.8	21.0	3/3	112	
UR A LO	37.9	37.4	38.4	32.4	0/3**	2	2
UR A MED	33.7	31.4	32.6	26.4	0/3**	1	
UR A HI	26.2	26.5	26.4	21.0	0/3**	2	
UR A optimized LO	32.1	31.9	32.0	30.1*	3/3**	25	27
UR A optimized MED	25.9	25.9	25.8	24.1*	3/3**	28	
UR A optimized HI	20.7	20.6	20.4	18.7*	3/3**	26	
UR B LO	33.3	33.2	33.8	32.4	2/3	49	52
UR B MED	27.4	27.3	27.4	26.4	3/3	49	
UR B HI	21.7	21.7	21.7	21.0	3/3	59	
UR B optimized LO	30.7	30.8	30.6	30.1*	3/3	65	58
UR B optimized MED	24.8	24.8	24.8	24.1*	3/3	60	
UR B optimized HI	19.5	19.5	19.9	18.7*	2/3	50	

* Expected Ct decreased by 2.3 due to 5-fold higher volume of spiked specimen (1 ml vs. 0.2 ml).
** Number of biological replicates within 2 Ct units from expected Ct.
Rep, biological replicate; WB, whole blood; UR, urine; LO, low spiking load; MED, medium spiking load; HI, high spiking load.
